# Case report and review of literature: Resection of a huge mediastinal low-grade fibromyxoid sarcoma with neck, axillary, and lung involvement

**DOI:** 10.3389/fsurg.2022.988881

**Published:** 2022-09-23

**Authors:** Natalie Khamashta, Ahmad Dalal, Mo’men Alashwas, Mayar Idkedek, Firas Abu-Akar

**Affiliations:** ^1^ Medical Research Club, Faculty of Medicine, Al-Quds University, Jerusalem, Palestine; ^2^ Department of Cardiothoracic Surgery, Al-Makassed Charitable Society Hospital, Jerusalem, Palestine

**Keywords:** low-grade fibromyxoid sarcoma, thoracotomy, surgical resection, huge sarcoma, mediastinum

## Abstract

Low-grade fibromyxoid sarcoma is an extremely rare malignant neoplasm, with an incidence of 0.18 per million, and comprises 0.6% of all soft tissue sarcomas. It has a high recurrence rate and late metastatic spread and is chemotherapy and radiotherapy insensitive. This paper reports a case of an unusually large mediastinal low-grade fibromyxoid sarcoma in a 55-year-old patient. The tumor was engulfing the main blood vessels of the mediastinum, involving the lung, and extending beyond the chest cavity to involve the cervical and axillary regions. The patient has a 21-year history of frequent surgical resections for lesions that were repeatedly misdiagnosed as neurofibroma. The tumor was successfully resected by a challenging operation that involved mediastinal mass resection, chest wall mass resection, and wedge resection of the left upper lobe of the lung. The deceivingly benign-looking histology of this tumor makes it a commonly misdiagnosed one, requiring careful assessment by pathologists to reach the right diagnosis. Surgical resection with clear margins remains the treatment of choice for these lesions. Due to the behavior of this tumor, once detected and managed, extensive long-term follow-up is always recommended.

## Introduction

Low-grade fibromyxoid sarcoma (LGFMS) is a rare soft tissue malignant tumor with relatively benign histological characteristics, late metastatic potential, and an alternating fibrous and myxoid background (1). Evans originally described it in 1987 (2), and Maretty-Nielsen et al. (3) in 2013 were the first to report the incidence of LGFMS, which was estimated to be 0.18 per million. Its histological features, including the characteristically scant cellularity, ample collagen, and bland cellular morphology, correspond to that of other benign and malignant tumors (1). These histological features resulted in LGFMS being a commonly misdiagnosed tumor, often mistaken for fibromatosis, malignant fibrous histiocytoma, and neurofibroma (1, 3). People of any age can be affected by LGFMS. The mean age of incidence is 33 years, and other estimations are around 40 years, with an approximately equal male-to-female ratio in adult cases (4–6).

Most LGFMS tumors are deep-seated lesions originating from soft tissues in the proximal limbs and torso; they usually grow slowly and present as a nontender mass. Grossly, the tumor has a well-defined white appearance. Microscopically, the cells have a spindle to stellate appearance, with small, angulated nuclei and little cytoplasm (1). Commonly, cells lack a particular arrangement or may be found organized in a whorled growth pattern. In fewer cases, a fascicular growth pattern might also be seen, usually associated with atypia and hypercellularity (1, 6).

Genetically, chromosomal translocations leading to fusion of the FUS and CREB3L2 genes are associated with most cases of LGFMS. FUS-CREB3L1 fusion is also reported with a lower incidence. However, some tumors present with neither translocations (1). In terms of immunohistochemistry, 100% of LGFMSs overexpress MUC4, whereas most other soft tissue tumors do not, making MUC4 a highly sensitive marker for diagnosis (7).

LGFMSs are distinguished by their high potential for metastatic spread and recurrence, with the lung being the most common site of metastasis. Owing to their low mitotic activity, LGFMSs are not considered sensitive to chemotherapy or radiotherapy and, therefore, are not used in treatment. Surgical excision remains the mainstay of treatment (1, 3).

Here, we present a case of a huge low-grade fibromyxoid sarcoma engorging the main mediastinal blood vessels with neck and axillary involvement and infiltration to the left lung, necessitating wedge resection of the upper lobe. The patient was complaining of dyspnea on mild exertion, orthopnea, and dysphagia to solids. This case was repeatedly misdiagnosed for over 21 years as a neurofibroma and was correctly identified only after her most recent resection surgery at our center. The patient’s treatment course included multiple resections over the mentioned period.

## Case description

A 55-year-old female patient was admitted to our hospital, complaining of shortness of breath on mild exertion of 5 months.

The patient’s history goes back 21 years ago when she noticed an anterior neck swelling that was not found to be associated with thyroid abnormalities. Tumor resection followed by radiotherapy was done, and paralysis of her left arm was noted afterward. Twelve years later, in 2013, she developed shortness of breath, for which she had a chest CT that showed a lobulated mediastinal mass, which was resected *via* median sternotomy. The acquired pathology report from that time indicated a neurofibroma. Three months later, she noticed a lateral neck mass of about 5 × 5 cm, which was resected.

Six years later, in 2019, she noticed a left axillary mass, and postresection pathology results were consistent with neurofibroma, with immunohistochemistry results negative for keratin and CD34 but focally positive for S100.

**Figure 1:**
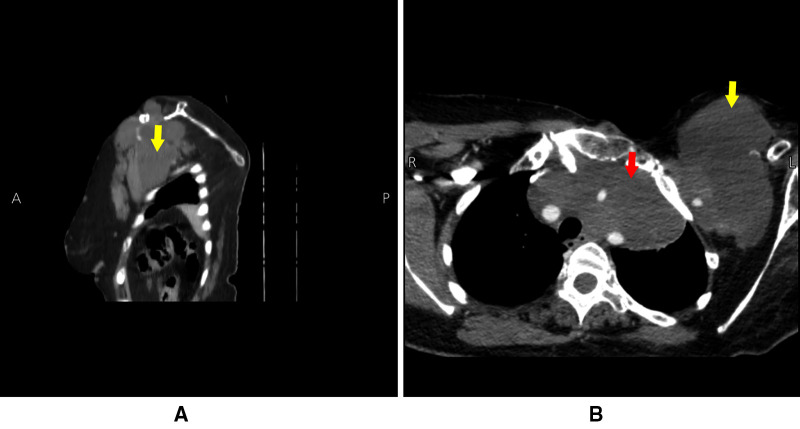
CT scans done in June 2021 showing a large lobulated hypodense mass lesion. Red arrow indicates the mediastinal lesion, yellow arrows indicate the extrathoracic chest wall lesion.

In June 2021, 3 months prior to her recent surgery, the patient complained of dyspnea on mild exertion, orthopnea, dysphagia to solids, and left arm pain and numbness. There was no history of weight loss, fever, or night sweats, and therefore, she underwent a chest CT with contrast, which showed a large lobulated hypodense mass in the left axillary region which involved suprascapular and clavicular components of about 7 × 10 × 15 cm. The mass was engulfing the major cervical vessels displacing the internal carotid arteries, elevating the left hemidiaphragm, and a necrotic supraclavicular lymph node was also noted at the base of the left side of the neck. In addition, an enhancing soft tissue mass on the right anterior chest wall indenting the second rib and widening neural foramina was seen (Figure 1).

In September 2021, the patient was admitted on the basis of her last CT scans and having recurrent lesions extending to the mediastinum with a history of four prior resections in addition to her complaints of shortness of breath on mild exertion and orthopnea.

The patient had a normal physical examination except for bilateral varicose veins and left upper arm paralysis. Multiple scars were noted on the neck and chest, indicating previous surgeries. Further examination revealed two left lateral cervical lesions, each measuring about 3 cm, in addition to a huge anterior lesion on the chest, extending to the left shoulder, causing loss of muscle mass and bone integrity, and associated with dilated superficial veins.

## Diagnostic assessment

Based on her history, physical examination, and prior CT scans, the patient was suspected of having a mediastinal neurofibroma and was scheduled for mediastinal mass resection, chest wall mass resection, and wedge resection of the left upper lobe of the lung.

**Figure 2:**
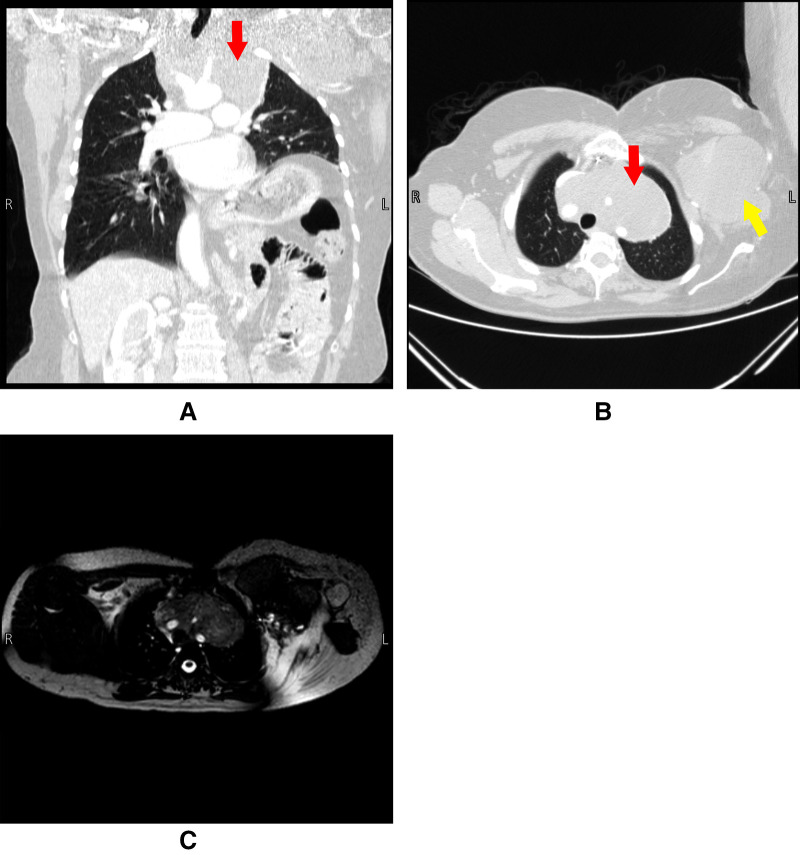
Panel (**A**) and (**B**) are CT scans done pre-operatively on 4/9/2021. Panel (**C**) is an MRI done pre-operatively on 8/9/2021. The figure shows a large anterior mediastinal mass engulfing the arch of the aorta and its branches. Red arrows indicate the mediastinal lesion, yellow arrow indicates the extrathoracic chest wall lesion.

Chest CT and whole spine MRI showed extension of the tumor into the anterior upper mediastinum encasing major vessels including the aorta, brachiocephalic trunk, bilateral carotids, and subclavian arteries. The brachiocephalic veins were also severely compressed, with the left being completely obstructed, resulting in multiple collateral veins in the left chest wall and axilla. Bone erosion of the first and second ribs and the clavicles was noted. The enhancing soft tissue mass on the right anterior chest wall did not undergo any significant changes (Figure 2).

Neck CT with and without contrast revealed a hypodense mass measuring 1.8 × 1 cm anterior to the left jugular vein, degenerative change in the cervical spine, and a few enlarged lymph nodes. Echocardiogram was normal, and the patient’s laboratory results were unremarkable.

### Surgery and postoperative course

Mass resection surgery was performed under general anesthesia in a supine position with antiseptic scrubbing and covering of the skin. After anesthesia, the patient was prepared to be connected to a cardiopulmonary bypass machine in case of emergency. This was done by preparing the groin, inserting arterial and venous femoral access lines, and priming the machine. A sternothoracotomy incision (open book incision) was done. The mass was found to be adherent to the aortic arch, its branches, and the phrenic nerve. The aortic arch was seen after the mediastinal dissection of the mass. The tumor was carefully resected over the aortic arch from right to left. The tumor wrapped the brachiocephalic artery at its origin, and the dissection of the tumor in the area surrounding it was successfully done, resulting in the freeing of the artery after securing it using a vessel loop.

The left common carotid and the left subclavian arteries were embedded in the tumor and therefore dissected and encircled using vessel loops. Resection of the tumor was done with both blunt digital and sharp scissors dissection.

After separating the mediastinal mass from the chest wall and aortic arch vessels, the invaded part of the left upper lobe of the lung was stapled. A decision was made for staged surgery to remove the extrathoracic lesion in another session. Hemopneumostasis was done, and a chest drain was inserted. The wound was closed in layers, and the patient was then transferred to the ICU, where she was stable on mechanical ventilation.

**Figure 3:**
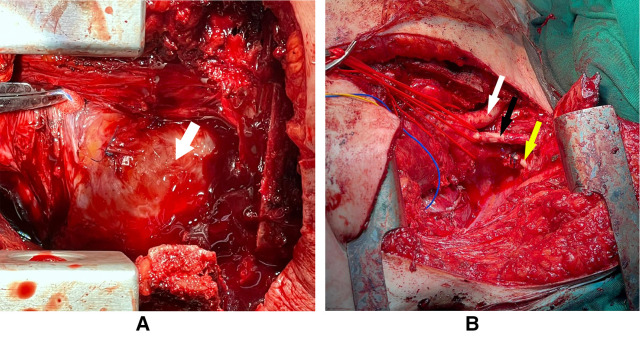
The arrow in panel (**A**) pointing at the tumor which is occupying the whole anterior mediastinum with some sutures from previous surgery. Panel (**B**) showing the arch of the aorta and its branches after the total resection of the intrathoracic tumor (white arrow: brachiocephalic artery, black arrow: left common carotid, yellow arrow: left subclavian artery).

Figure 3 shows the intraoperative pictures of the mediastinum before and after tumor resection. The postoperative course was complicated by chylothorax, which was successfully treated conservatively by a low-fat diet and total parenteral nutrition, in addition to a superficial wound infection that was treated with proper antibiotics.

Follow-up CT scans revealed the extrathoracic parts of the tumor, and as of the publication date, the patient is scheduled for further surgery to resect them.

**Figure 4:**
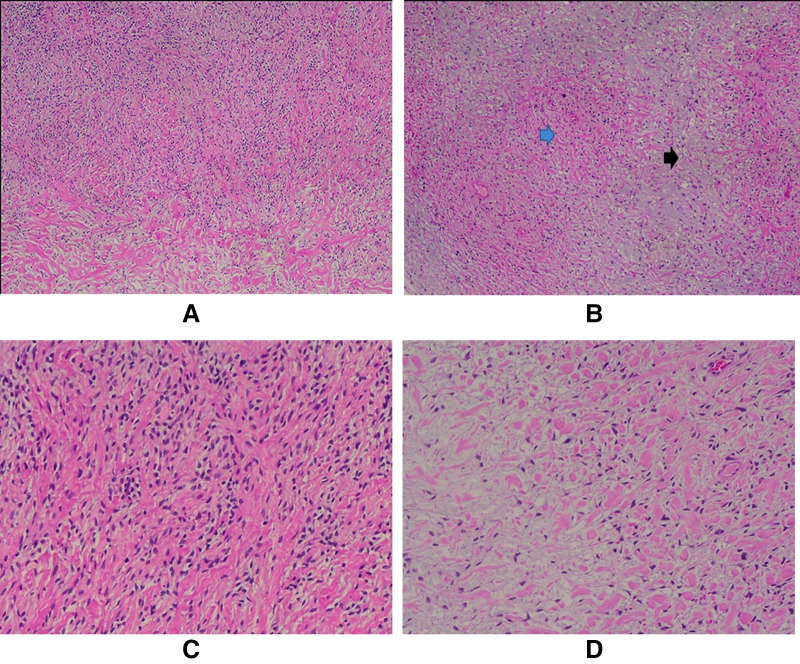
**A**. Mild to moderately cellular tumor (H/E, 4X); **B**. The biphasic growth pattern is demonstrated with both fibrous (blue arrow) and myxoid (black arrow) areas (H/E, 4X); **C**. The tumor cells are mostly bland oval to spindle-shaped (H/E, 10X) and stellate (**D**); (H/E, 10X).

The chest drains were removed on day 15 after the surgery, and the patient was discharged on day 20 postop in very well condition. Her medication list included acetylcysteine, furosemide, paracetamol, esomeprazole, and bisoprolol. Pathology results were consistent with a low-grade fibromyxoid sarcoma. By immunohistochemistry, tumor cells were diffusely positive for BCL2 and negative for CK7, S100, CD34, STAT6, MDM2, SMA, and beta-catenin. Grossly, the tumor cut surface was tan, white, and homogenous, with parts of it appearing mucoid. Microscopic analysis showed a biphasic tumor of mild to moderate to focally high cellularity set in fibrous and myxoid background. The tumor cells were mostly bland oval to stellate to spindle; focal areas showed increased nuclear atypia, and infarction-type necrosis was noted. Mitotic activity was not increased (Figure 4). An overview of the course of diagnosis, treatment and management is shown in the Timeline table (Supplementary Material) section.

The tumor was resected in pieces, the three largest of which measured 16 × 15 × 2, 10 × 7.5 × 4, and 9 × 2.5 × 1 cm. Other smaller pieces measured between 1 and 4 cm.

### Timeline table

**Table:** 

Date	Event
2000	The patient noticed an anterior neck swelling and underwent tumor resection followed by radiotherapy
September/2013	The patient developed shortness of breath, and her chest CT showed mediastinal lobulated lesions that were resected *via* median sternotomy
December/2013	A lateral neck mass was noticed and resected
October/2019	The patient noticed a left axillary mass that was also resected
June/2021	Chest CT scan: a large lobulated hypodense mass in the left axillary region with involved suprascapular and clavicular components of about 7 × 10 × 15 cm. The mass has superior mediastinum continuity engulfing the major cervical vessels
September/2021	The patient was admitted and scheduled for mediastinal mass resection, chest wall mass resection, and wedge resection of the left upper lobe of the lung
September/2021	– Chest CT scan: extension of the tumor into the anterior upper mediastinum encasing major vessels, and multiple collateral veins were noticed on the left side
	– Neck CT scan: a hypodense mass anterior to the left jugular vein
September/2021	Mass resection surgery was performed, and a decision for staged surgery was made
September/2021	– The postoperative course was complicated by chylothorax and a superficial wound infection, which were successfully treated
	– Follow-up CT scans revealed the extrathoracic parts of the tumor
October/2021	– The patient was discharged in very good condition

## Discussion

Fibromyxoid sarcomas represent 0.6% of all soft tissue sarcomas (3). LGFMSs usually arise in the proximal extremities or the trunk but can rarely arise at other locations, including the head, neck, bowels, and other less common sites (1).

On gross examination, LGFMSs can be as small in size as 1 cm or extend up to 20 cm (1). To our knowledge, nine other cases of mediastinal LGFMSs were reported in the literature, with sizes ranging from 8 to 23.5 cm. Table 1 presents a summary table of all the reported cases of mediastinal LGFMS (8–15). In our case, it was difficult to estimate the gross size of the tumor due to its extensive growth pattern. Although half-resected and in pieces, one of the individual pieces of the tumor measured up to 16 × 15 × 2 cm in size, with the other two large pieces measuring about 10 cm each, in addition to other smaller fragments. Macroscopically, LGFMS appears as a well-defined mass that has a characteristic white fibrous cut surface usually interspersed with focal myxoid areas, which is consistent with our case (1).

**Table 1:** Reported cases of mediastinal low-grade fibromyxoid sarcoma.

Reference	Age/gender	Site	Intervention	Size (cm)	Recurrence
Takanami et al. (9), 1999, Japan	35, male	Anterior mediastinum	Surgical resection	9 × 5.5 × 3	Recurrence after 9 years
Galetta et al. (10), 2004, Italy	41, male	Anterior mediastinum, posterior to the thymus	Surgical resection followed by radiation	8 × 4 × 3	No recurrence after 35 months
Jakowski and Wakely (11), 2008, USA	44, female	Right side of the epicardium	Surgical resection	12	No recurrence within 7 months
Maeda et al. (12), 2009, Japan	19, female	Anterior mediastinum	Surgical resection	23.5 × 10 × 21.5	Recurrence after 5 years
Maeda et al. (12), 2009, Japan	50, male	Right superior mediastinum	Surgical resection	13 × 13 × 13	No recurrence after 5 years
Gülhan et al. (8), 2012, Turkey	25, female	Posterior mediastinum	Surgical resection	17 × 13 × 11	Not reported
Perez et al. (13), 2019, USA	32, male	Parietal pleura origin	Surgical resection	11	Not reported
Sajid et al. (15), 2021, Pakistan	26, male	Anterior mediastinum	Surgical resection	17 × 12 × 11	No recurrence after 22 months
Williams et al. (14), 2021, USA	50, male	Adjacent to the pericardium	Doxorubicin	Not reported	Not reported

Histology-wise, LGFMSs are either infiltrative or circumscribed, with bland-looking spindle cells in a mixed fibromyxoid background. Most tumors typically show low to moderate cellularity, with mitotic figures being usually scant or undetectable. The myxoid areas of the tumor are usually found to have a curvilinear network of capillaries (1, 6).

LGFMSs can present with a diverse range of unusual or high-grade histological features; some of them were present in our patient’s histological findings, most prominently, increased cellularity, nuclear atypia, and tumor necrosis. Other atypical features were not necessarily present in our case, including calcification, ossification, multinucleated giant cells, and other changes. Such features are not considered of prognostic value, nor have they been associated with tumor behavior (1, 6).

Our patient’s condition was misdiagnosed as a neurofibroma until her last surgery. Neurofibromas are a common misdiagnosis for LGFMSs among other neoplasms due to similar histological features. Neurofibromas are benign tumors of the peripheral nerve sheath characterized by the random arrangement of their spindled cells in a myxoid background, in addition to the presence of thin wispy or thick bulky collagenous fibers (16). In contrast, LGFMSs lack some of the characteristic histological features of neurofibromas, such as mast cells and the elongated undulating nerve sheath-like nuclei. Neurofibromatous tumors are relatively consistent with no sharp changes from fibrous to myxoid zones and no prominent curvilinear vasculature, both of which are seen in LGFMS. S100 and CD34 as tumor markers are commonly found to be negative in LGFMS, whereas neurofibromas usually express positive reactivity. Our patient’s pathology report from 2019 reported focal positivity for S100, which indicates that LGFMS can sometimes express this protein and therefore lead to misdiagnosis (1).

MUC4 gene is overexpressed in LGFMSs in addition to FOXL1 and CD34. MUC4, which is a high-molecular-weight transmembrane glycoprotein, remains the most sensitive marker for diagnosing LGFMSs (7). Some other tumor markers may be overexpressed, such as BCL-2, CD99, vimentin, and epithelial membrane antigen (17). However, these are of less diagnostic value due to their low specificity (1). Rarely, other markers may be focally positive, such as smooth muscle actin, CD34, desmin, and cytokeratin. LGFMSs are most frequently negative for S100 protein, GFAP, h-caldesmon, beta-catenin, MDM2, and CD117 (17).

The mainstay of treatment of LGFMSs is surgical resection with clear margins. Other surgical approaches such as local excision, radical surgery, wide *en bloc* resection, and compartmental resection were reported in the literature. Surgery is the only treatment that can result in disease-free periods (3, 18).

This kind of surgery is very challenging but still doable. Surgeons can usually find the cleavage line and the proper plane for dissecting the tumor. The key to safe dissection in this patient’s case was to find the arch of the aorta, peel the tumor off it, and then continue upward in the same plane. The surgeon should be ready for the worst scenarios; the heart lung bypass machine should be ready and primed, femoral access should be prepared before the surgery, and lines for the cardiopulmonary bypass machine should be inserted before the operation.

In our case, a staged surgical strategy was followed for two main reasons: the primary target of the surgery was to release the mediastinal compression from the heart and the great vessels, which was risking the patient’s life, and this was accomplished with a complex and very long operation. Dealing with the extrathoracic lesion in the same session would make it even more complex and prolonged. Another reason for this decision is the slow-growing nature of this tumor, making it possible to give the patient some time to recover with reasonable risk before undergoing any further surgical intervention.

The use of chemotherapy and radiotherapy as a stand-alone or adjuvant treatment for LGFMS remains unclear, with many studies showing that it merely slows disease progression. Chemotherapeutic agent trabectedin has been shown to be effective in translocation-related soft tissue sarcomas. However, its role in the treatment of LGFMS remains unclear (3, 18, 19).

Ideally, long-term management requires a rigorous follow-up involving whole-body scans. According to Maretty-Nielsen et al. (3), the best recommendation to scan for and diagnose LGFMSs is a full-body MRI scan to detect any disseminated lesions. MRI scans have shown higher sensitivity than PET-CTs, as the latter may not detect lesions and delay proper management (3).

## Conclusion

Despite their benign-appearing histology, low-grade fibromyxoid sarcomas are malignant tumors characterized by the high possibility of local recurrence in addition to metastasis, with most patients requiring multiple surgeries in their lifetime. This necessitates good diagnostic criteria to correctly identify the malignant lesions of LGFMS among other similar-presenting lesions that may direct the physician to follow a different course of treatment. Early detection and diagnosis lead to better outcomes with regard to complications and possible metastasis. Once diagnosed, proper and long-term follow-up using full-body scanning technologies, ideally using MRI, is required to detect any new lesions early in their course and improve outcomes. To our knowledge, no treatment protocol has been established for treating LGFMSs, and studies are still being conducted to determine the efficacy of chemotherapeutic agents and radiotherapies on these tumors. Because LGFMS can be somewhat challenging to diagnose in addition to being very rare, pathologists should be able to recognize the histology of LGFMS better and recommend the appropriate immunohistochemical tests to be certain of their final diagnosis.

## Patient perspective

LGFMS patients suffer recurrent symptoms every few years, among which are compression symptoms caused by the size of the tumor and its malignant tendencies that may cause infiltration to other organs and therefore create an array of possible symptoms. LGFMSs’ tendency to recur requires patients to undergo multiple surgeries over the years, which might inflict on their quality of life, and take a mental toll on them and their families. Long-term follow-up might also be inconvenient for patients from a financial perspective and in terms of access and availability.

## Data Availability

The original contributions presented in the study are included in the article/Supplementary Material, further inquiries can be directed to the corresponding author.

## References

[1] Mohamed M Fisher C Thway K. Low-grade fibromyxoid sarcoma: clinical, morphologic and genetic features. Ann Diagn Pathol. (2017) 28:60–7. 10.1016/j.anndiagpath.2017.04.001 28648941

[2] Evans HL. Low-grade fibromyxoid sarcoma: a report of two metastasizing neoplasms having a deceptively benign appearance. Am J Clin Pathol. (1987) 88(5):615–9. 10.1093/ajcp/88.5.615 3673943

[3] Maretty-Nielsen K Baerentzen S Keller J Dyrop HB Safwat A. Low-grade fibromyxoid sarcoma: incidence, treatment strategy of metastases, and clinical significance of the *FUS* gene. Sarcoma. (2013) 2013:1–6. 10.1155/2013/256280 PMC3683502 23818812

[4] Rekhi B Deshmukh M Jambhekar NA. Low-grade fibromyxoid sarcoma: a clinicopathologic study of 18 cases, including histopathologic relationship with sclerosing epithelioid fibrosarcoma in a subset of cases. Ann Diagn Pathol. (2011) 15(5):303–11. 10.1016/j.anndiagpath.2011.02.005 21550274

[5] Mertens F Fletcher CDM Antonescu CR Coindre JM Colecchia M Domanski HA Clinicopathologic and molecular genetic characterization of low-grade fibromyxoid sarcoma, and cloning of a novel FUS/CREB3L1 fusion gene. Lab Invest. (2005) 85(3):408–15. 10.1038/labinvest.3700230 15640831

[6] Folpe AL Lane KL Paull G Weiss SW. Low-grade fibromyxoid sarcoma and hyalinizing spindle cell tumor with giant rosettes: a clinicopathologic study of 73 cases supporting their identity and assessing the impact of high-grade areas. Am J Surg Pathol. (2000) 24(10):1353–60. 10.1097/00000478-200010000-00004 11023096

[7] Doyle LA Möller E Dal Cin P Fletcher CDM Mertens F Hornick JL. MUC4 is a highly sensitive and specific marker for low-grade fibromyxoid sarcoma. Am J Surg Pathol. (2011) 35(5):733–41. 10.1097/PAS.0b013e318210c268 21415703

[8] Gülhan SŞ. Low-grade fibromyxoid sarcoma in the mediastinum: a case report. Turkish Journal of Thoracic and Cardiovascular Surgery. (2012) 20(2). 10.5606/tgkdc.dergisi.2012.073

[9] Takanami I Takeuchi K Naruke M. Low-grade fibromyxoid sarcoma arising in the mediastinum. J Thorac Cardiovasc Surg. (1999) 118(5):970–1. 10.1016/S0022-5223(99)70076-0 10534712

[10] Galetta D Cesario A Margaritora S Granone P. Primary mediastinal hyalinizing spindle cell tumor with giant rosettes. Ann Thorac Surg. (2004) 77(6):2206–9. 10.1016/S0003-4975(03)01388-2 15172307

[11] Jakowski JD Wakely PE. Primary intrathoracic low-grade fibromyxoid sarcoma. Hum Pathol. (2008) 39(4):623–8. 10.1016/j.humpath.2007.08.017 18275982

[12] Maeda E Ohta S Watadani T Goto A Nakajima A Ohtomo K. Imaging findings of thoracic low-grade fibromyxoid sarcoma: report of three cases. Jpn J Radiol. (2009) 27(9):375–80. 10.1007/s11604-009-0351-2 19943150

[13] Perez D El-Zammar O Naous R. Low-grade fibromyxoid sarcoma: a rare case in an unusual location. Am J Clin Pathol. (2019) 152(Supplement_1):S54. 10.1093/ajcp/aqz113.042 PMC7436804 32874586

[14] Williams CM Du W Mangano WE Mei L. Mediastinal low-grade fibromyxoid sarcoma with FUS-CREB3L2 gene fusion. Cureus. (2021) 13(6):e15606. 10.7759/cureus.15606. Available from: https://www.cureus.com/articles/59562-mediastinal-low-grade-fibromyxoid-sarcoma-with-fus-creb3l2-gene-fusion (Accessed August 11, 2022). 34277226 PMC8273027

[15] Sajid MI Arshad S Abdul-Ghafar J Fatimi SH Din NU. Low-grade fibromyxoid sarcoma incidentally discovered as an asymptomatic mediastinal mass: a case report and review of the literature. J Med Case Reports. (2021) 15:50. 10.1186/s13256-020-02605-4 PMC7851906 33526082

[16] Messersmith L Krauland K. Neurofibroma. In: Statpearls [Internet]. Treasure Island, FL: StatPearls Publishing (2022). Available from: http://www.ncbi.nlm.nih.gov/books/NBK539707/ (Accessed February 19, 2022).

[17] Guillou L Benhattar J Gengler C Gallagher G Ranchère-Vince D Collin F Translocation-positive low-grade fibromyxoid sarcoma: clinicopathologic and molecular analysis of a series expanding the morphologic spectrum and suggesting potential relationship to sclerosing epithelioid fibrosarcoma: a study from the French Sarcoma Group. Am J Surg Pathol. (2007) 31(9):1387–402. 10.1097/PAS.0b013e3180321959 17721195

[18] Toro C Costa P Vecchio GM Magro G. Low-grade fibromyxoid sarcoma of the parapharyngeal space: a case report and review of the literature. Oral Maxillofac Surg Cases. (2020) 6(2):100152. 10.1016/j.omsc.2020.100152

[19] Le Cesne A Cresta S Maki RG Blay JY Verweij J Poveda A A retrospective analysis of antitumour activity with trabectedin in translocation-related sarcomas. Eur J Cancer. (2012) 48(16):3036–44. 10.1016/j.ejca.2012.05.012 22749255

